# A high-efficiency geminivirus-based vector system for rapid gene function characterization and transient overexpression in *Cucurbita* species

**DOI:** 10.1093/hr/uhag200

**Published:** 2026-05-13

**Authors:** Chao Geng, Yan-Di Cheng, Si-Ru Shi, Ling-Zhi Liu, Xiao Yin, Shan-Shan Jiang, Jun Jiang, Zhi-Yong Yan, Yan-Ping Tian, Xiang-Dong Li

**Affiliations:** Department of Plant Pathology, College of Plant Protection, Shandong Agricultural University, Tai’an, Shandong 271018, China; Department of Plant Pathology, College of Plant Protection, Shandong Agricultural University, Tai’an, Shandong 271018, China; Department of Plant Pathology, College of Plant Protection, Shandong Agricultural University, Tai’an, Shandong 271018, China; Department of Plant Pathology, College of Plant Protection, Shandong Agricultural University, Tai’an, Shandong 271018, China; Institute of Plant Protection, Shandong Academy of Agricultural Sciences, Ji’nan, China; Institute of Plant Protection, Shandong Academy of Agricultural Sciences, Ji’nan, China; Department of Plant Pathology, College of Plant Protection, Shandong Agricultural University, Tai’an, Shandong 271018, China; Department of Plant Pathology, College of Plant Protection, Shandong Agricultural University, Tai’an, Shandong 271018, China; Department of Plant Pathology, College of Plant Protection, Shandong Agricultural University, Tai’an, Shandong 271018, China; Department of Plant Pathology, College of Plant Protection, Shandong Agricultural University, Tai’an, Shandong 271018, China; Institute of Plant Protection, Shandong Academy of Agricultural Sciences, Ji’nan, China

Dear Editor,

Species of the genus *Cucurbita*, including *Cucurbita pepo* (zucchini) and *Cucurbita moschata* (squash), comprise economically important vegetable crops [1]. Despite the release of reference for several *Cucurbita* species, effective reverse genetic tools for rapid gene function analysis in these crops remain underdeveloped. Virus-induced gene silencing (VIGS) and virus-induced gene overexpression (VIGO) are powerful reverse genetics tools that enable rapid functional characterization of plant genes and directed evolution of target genes [2–4]. However, VIGS and VIGO vector systems have not yet been established in *Cucurbita* species. Here, we developed VIGS and VIGO vectors based on squash leaf curl China virus (SLCCNV), a bipartite DNA virus in the family Geminiviridae, for rapid gene function characterization in *Cucurbita* species.

The genome of SLCCNV consists of two circular single-stranded circular DNA components. An infectious clone of SLCCNV isolate from WeiFang, Shandong Province, China was assembled in the binary vector pCB301, generating pSLCCNV-A (harboring 1.4-mer tandem repeats of DNA-A) and pSLCCNV-B (harboring 1.5-mer tandem repeats of DNA-B) (Fig. 1A, Tables S1 and S2). *Agrobacterium tumefaciens* GV3101 cultures carrying pSLCCNV-A and pSLCCNV-B were co-infiltrated into leaves of *C. pepo* and *C. moschata*. At 12 days post-agroinfiltration (dpai), inoculated plants developed characteristic leaf-curl symptoms, accompanied by robust accumulation of SLCCNV genomic DNA in newly emerged upper leaves (Fig. 1B).

**Figure 1:**
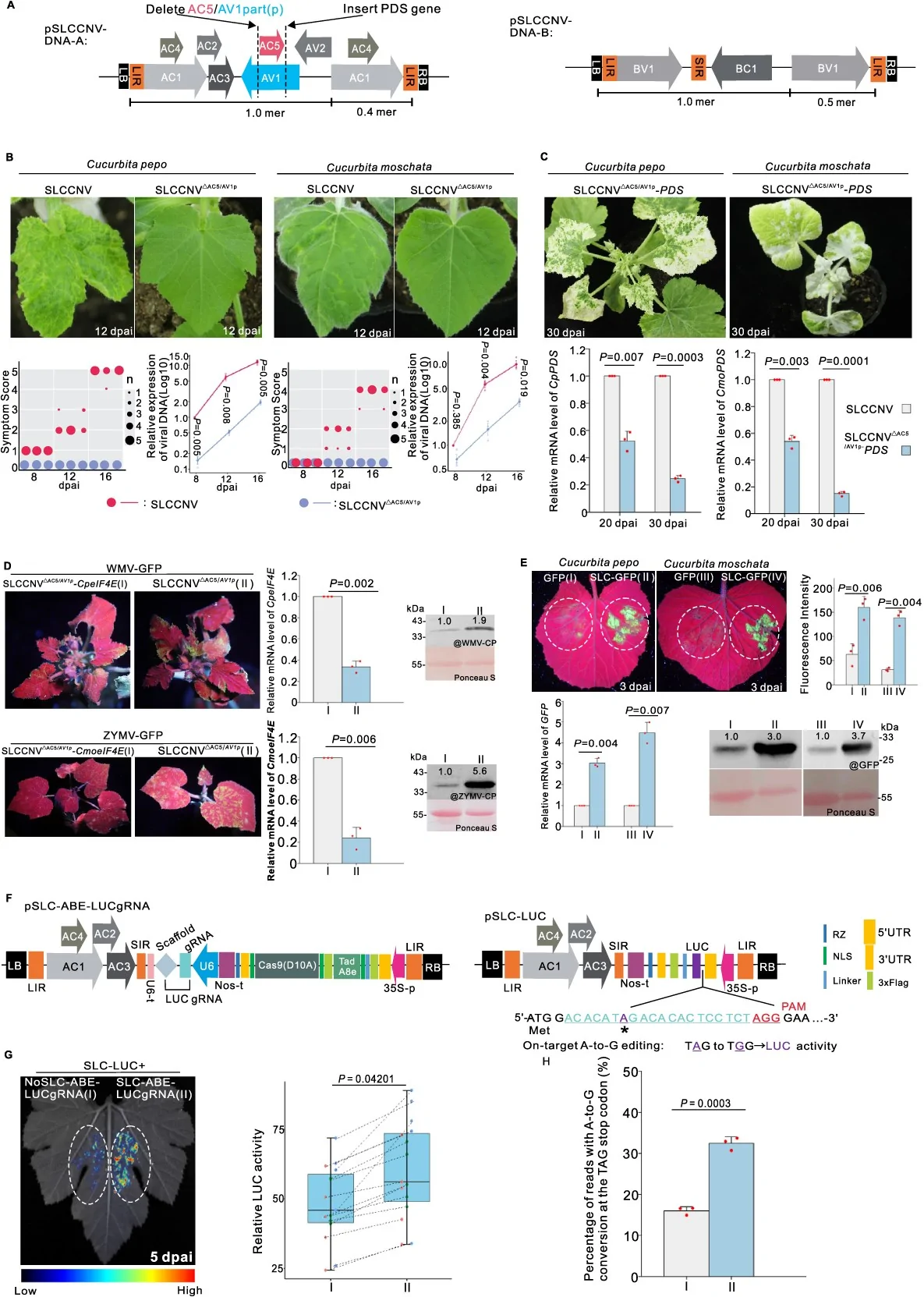
SLCCNV-based vectors for gene silencing and overexpression in *Cucurbita* species. (A) Schematic representation of the SLCCNV-derived vectors. DNA-A encodes AC1–AC5, AV1, and AV2, and DNA-B encodes BV1 and BV2. LIR, long intergenic region; SIR, short intergenic region. (B) Phenotype and RT-qPCR detection of SLCCNV- and SLCCNV^△AC5/AV1p^-infected *C. pepo* and *C. moschata* plants. (C) Phenotype and RT-qPCR analysis of *PDS*-silenced *C. pepo* and *C. moschata* plants. (D) Fluorescence imaging, RT-qPCR, and western blot analysis of WMV-GFP and ZYMV-GFP infected *eIF4E*-silenced and control *C. pepo* and *C. moschata* plants at 10 days post-inoculation. (E) Fluorescence, RT-qPCR, and western blot analysis of GFP and SLC-GFP in *C. pepo* and *C. moschata* leaf patches at 3 dpai. (F) Schematic diagram of SLCCNV-based vectors. (G) LUC-based chemiluminiscence of pSLC-ABE- and pNoSLC-ABE-agroinfiltrated patches in *C. pepo* leaves at 5 dpai. (H) Percentage of reads showing A-to-G conversion at the introduced TAG stop codon in the LUC sequence. Statistical analysis was carried out using two-sided Student’s *t*-tests for B–E and G–H. RT-qPCR and fluorescence intensity data are presented as the mean of three independent biological replicates, each with three technical replicates. All experiments were repeated at least three times with similar results. All plants were grown at 25°C/22°C (day/night) under a 16 h photoperiod. *A. tumefaciens* GV3101 cultures were adjusted to an optical density at 600 nm (OD_600_) of 0.5 before infiltration.

To attenuate the severe symptoms induced by wild-type SLCCNV, we deleted the virulence-associated *AC5* gene and a portion of the *AV1* gene (AV1p) overlapping with *AC5*. A previous study reported that loss of these genes does not compromise the systemic infection of bipartite geminiviruses [5]. The resulting construct was designated pSLCCNV^△AC5/AV1p^ (Fig. 1A). This mutant did not induce obvious viral symptoms (Fig. 1B and Fig. S1). Despite accumulating lower levels of viral DNA than wild-type SLCCNV (Fig. 1B), it maintained systemic movement and was therefore evaluated for its utility in VIGS. A 300-bp fragment of the *C. pepo PHYTOENE DESATURASE* (*CpPDS*) gene was inserted into pSLCCNV^△AC5/AV1p^ to generate pSLCCNV^△AC5/AV1p^-*CpPDS*. Co-infiltration with pSLCCNV-B into *C. pepo* and *C. moschata* leaves induced *PDS* gene silencing in the systemically infected leaves at 20 dpai (Fig. 1C). By 30 dpai, the silencing efficiency reached ~70% in *C. pepo* and 90% in *C. moschata* (Fig. 1C). Next, the potyvirus susceptible gene eukaryotic initiation factor 4E (*eIF4E*) was targeted. Two 300-bp fragments of *CpeIF4E* and *CmoeIF4E* were individually inserted into pSLCCNV^△AC5/AV1p^. At 30 dpai, real-time quantitative PCR (RT-qPCR) analysis revealed significant reductions in *eIF4E* mRNA levels of ~70% in *C. pepo* and 85% in *C. moschata* compared with the empty vector controls (Fig. 1D). To assess the functional impact of *eIF4E* silencing, green fluorescent protein (GFP) tagged watermelon mosaic virus (WMV) and zucchini yellow mosaic virus (ZYMV) were mechanically inoculated into the *eIF4E*-silenced or control plants. At 10 days post-inoculation, GFP fluorescence was markedly weaker in *eIF4E*-silenced plants than in control plants, indicating that knockdown *eIF4Es* restrains WMV and ZYMV infection in both *C. pepo* and *C. moschata* (Fig. 1D). Western blot detection of the viral coat protein further confirmed significantly reduced WMV or ZYMV accumulation in the *eIF4E*-silenced plants (Fig. 1D). Consistently, these results establish SLCCNV as an effective VIGS vector for functional genomics in *Cucurbita* species.

Next, we engineered the SLCCNV infectious clone into a VIGO vector. The sequences encoding the SLCCNV replication-associated proteins AC1–AC4 together with the intergenic spacers were inserted into the binary vector pCambia1300 to generate replicon pSLC (Fig. S2). A 35S::GFP expression cassette was subsequently introduced into pSLC to obtain pSLC-GFP, whereas a nonreplicating control construct containing the same 35S::GFP expression cassette (pNoSLC-GFP) was generated in pCambia1300 (Fig. S2). At 3 dpai, the fluorescence intensity in pSLC-GFP-infiltrated patches was ~2.5-fold and 4.5-fold higher than in pNoSLC-GFP patches in *C. pepo* and *C. moschata* leaves, respectively (Fig. 1E). RT-qPCR analysis corroborated these observations, showing GFP transcript levels in pSLC-GFP patches were ~3.0-fold and 4.5-fold higher than those in the corresponding controls in *C. pepo* and *C. moschata*, respectively. Consistently, immunoblot analysis revealed ~3.0-fold and 3.7-fold increase in GFP accumulation in *C. pepo* and *C. moschata*, respectively (Fig. 1E). Together, these data demonstrate that the SLCCNV-derived replicon pSLC drives robust protein accumulation through replicon-mediated DNA amplification and subsequent enhanced transcription.

Finally, the utility of the SLCCNV-based system for gene editing was evaluated using a luciferase (LUC) reporter assay in which an adenine base editor (ABE) converts a premature TAG stop codon to TGG, thereby restoring LUC activity (Fig. 1F). Utilizing the pSLC replicon to enhance ABE expression, pSLC-ABE-infiltrated patches showed ~1.2-fold higher LUC activity than pNoSLC-ABE patches in *C. pepo* leaves (Fig. 1G). To confirm this result, the target *LUC* fragments were amplified from infiltrated leaf tissues and subjected to high-throughput sequencing. Amplicon sequencing revealed that the A-to-G conversion frequency at the engineered TAG stop codon was ~2-fold higher in pSLC-ABE patches than in pNoSLC-ABE patches (Fig. 1H and Table S3). These results demonstrate that SLCCNV-based vector can effectively enhance single-base editing efficiency *in planta*.

In summary, SLCCNV-based VIGS and VIGO vectors that enable rapid and effective gene knockdown, overexpression, and base editing in *Cucurbita* species were established. Most geminiviruses rely on phloem-specific promoters to drive transcription from their circular genomic DNA, restricting infection predominantly to phloem tissues and thereby limiting their usefulness as broad-tissue expression or silencing vectors. By contrast, SLCCNV systemically infects non-phloem tissues as demonstrated by the extensive SLCCNV^△AC5/AV1p^-*PDS*-induced photobleaching phenotype (Fig. 1C), making it particularly amenable to vector development in *Cucurbita*. With the growing use of virus-induced gene regulation systems for functional genomics in horticultural crops, the SLCCNV-based platforms described here are expected to accelerate functional validation of genes underlying agronomic trait genes and to facilitate dissection of regulatory pathways in *Cucurbita* species.

## Data Availability

The data underlying this article will be shared on reasonable request to the corresponding author. Supplementary Figures and Tables are available at Zenodo repository (https://zenodo.org/records/18751558).
